# Limited Impact of Passive Non-Neutralizing Antibody Immunization in Acute SIV Infection on Viremia Control in Rhesus Macaques

**DOI:** 10.1371/journal.pone.0073453

**Published:** 2013-09-09

**Authors:** Taku Nakane, Takushi Nomura, Shoi Shi, Midori Nakamura, Taeko K. Naruse, Akinori Kimura, Tetsuro Matano, Hiroyuki Yamamoto

**Affiliations:** 1 AIDS Research Center, National Institute of Infectious Diseases, Tokyo, Japan; 2 The Institute of Medical Science, The University of Tokyo, Tokyo, Japan; 3 Department of Molecular Pathogenesis, Medical Research Institute, Tokyo Medical and Dental University, Tokyo, Japan; Seattle Biomedical Research Institute, United States of America

## Abstract

**Background:**

Antiviral antibodies, especially those with neutralizing activity against the incoming strain, are potentially important immunological effectors to control human immunodeficiency virus (HIV) infection. While neutralizing activity appears to be central in sterile protection against HIV infection, the entity of inhibitory mechanisms via HIV and simian immunodeficiency virus (SIV)-specific antibodies remains elusive. The recent HIV vaccine trial RV144 and studies in nonhuman primate models have indicated controversial protective efficacy of HIV/SIV-specific non-neutralizing binding antibodies (non-NAbs). While reports on HIV-specific non-NAbs have demonstrated virus inhibitory activity *in vitro*, whether non-NAbs could also alter the pathogenic course of established SIV replication *in vivo*, likewise via neutralizing antibody (NAb) administration, has been unclear. Here, we performed post-infection passive immunization of SIV-infected rhesus macaques with polyclonal SIV-specific, antibody-dependent cell-mediated viral inhibition (ADCVI)-competent non-NAbs.

**Methods and Findings:**

Ten lots of polyclonal immunoglobulin G (IgG) were prepared from plasma of ten chronically SIV_mac239_-infected, NAb-negative rhesus macaques, respectively. Their binding capacity to whole SIV_mac239_ virions showed a propensity similar to ADCVI activity. A cocktail of three non-NAb lots showing high virion-binding capacity and ADCVI activity was administered to rhesus macaques at day 7 post-SIV_mac239_ challenge. This resulted in an infection course comparable with control animals, with no significant difference in set point plasma viral loads or immune parameters.

**Conclusions:**

Despite virus-specific suppressive activity of the non-NAbs having been observed *in vitro*, their passive immunization post-infection did not result in SIV control *in vivo*. Virion binding and ADCVI activity with lack of virus neutralizing activity were indicated to be insufficient for antibody-triggered non-sterile SIV control. More diverse effector functions or sophisticated localization may be required for non-NAbs to impact HIV/SIV replication *in vivo*.

## Introduction

Development of a successful vaccine is crucial for global human immunodeficiency virus (HIV) control. A recent clinical trial in Thailand has shown partial efficacy of an HIV vaccine regimen, RV144 [1]. Further analyses have suggested possible contribution of virus-binding antibodies to the protection from HIV infection [2,3]. Thus, understanding of the effect of virus-binding, non-neutralizing antibody (non-NAb) responses on the course of HIV/SIV infection may serve as one step for vaccine development.

In contrast to the constrained emergence of neutralizing antibodies (NAbs), non-NAbs are commonly induced in both the acute and chronic phase of HIV/SIV infection [4–6]. They are known to exhibit *in vitro* suppressive effects against virus replication, such as ADCC (antibody-dependent cellular cytotoxicity) and ADCVI (antibody-dependent cell-mediated virus inhibition) [7–10]. While several reports have suggested inverse correlation between such effector functions and viral loads in HIV-infected individuals [5,6] and vaccinated SIV-infected macaques [11–14], the precise influence of non-NAb responses on viral replication control remains undetermined. Passive immunization studies in nonhuman primate AIDS models have shown partial protection from mucosal virus challenge by mucosal pre-challenge non-NAb infusion, suggesting limited protective efficacy of locally-distributed non-NAb responses [15,16]. In the present study, we focused on the effect of systemic distribution of non-NAbs on established primary viral infection, which is another practical vaccine correlate.

Passive immunization of polyclonal neutralizing antibodies (NAbs), which does not exclude coexistence of non-NAbs, has partially provided protective activity in nonhuman primate AIDS models [17–19]. Additionally, we have reported SIV control *in vivo* by post-infection administration of polyclonal NAbs, in which enhanced antigen presentation and subsequent augmented T-cell responses likely accounted for the control [20,21]. Since non-NAbs are potentially capable of supporting these suggested mechanisms, the protective activity of non-NAbs by themselves against established primary infection is important to be assessed. Here, we examined the effect of passive non-NAb immunization at day 7 post-challenge on primary SIV_mac239_ replication in rhesus macaques. Despite the virion-binding and ADCVI activity of non-NAbs having been confirmed *in vitro*, passive immunization of non-NAbs did not result in control of SIV replication *in vivo*. 

## Methods

### Ethics Statement

Animal experiments were carried out in National Institute of Biomedical Innovation (NIBP) after approval by the Committee on the Ethics of Animal Experiments of NIBP in accordance with the guidelines for animal experiments at NIBP and National Institute of Infectious Diseases. To prevent viral transmission, animals were housed in individual cages allowing them to make sight and sound contact with one another, where the temperature was kept at 25 ^o^C with light for 12 hours per day. Animals were fed with apples and commercial monkey diet (Type CMK-2, Clea Japan, Inc.). Blood collection, vaccination, and SIV challenge were performed under ketamine anesthesia. Three of eleven macaques, R10-001, R10-004, and R06-029, were euthanized during the observation period in the SIV challenge experiment of this study. Two of them (R10-004 and R06-029) were euthanized (at 7-10 months) after the minimum observation period required for this study (6 months) because of the limitation of available cage numbers. One macaque R10-001 was euthanized (at 9 months) at the endpoint for euthanasia, which was determined by typical signs of AIDS including reduction in peripheral CD4^+^ T-cell counts (less than 200 cells/µl), 10% loss of body weight, diarrhea, and general weakness. At euthanasia, animals were deeply anesthetized with pentobarbital under ketamine anesthesia, and then, whole blood was collected from left ventricle.

### Analysis of Virus-Specific Neutralizing Responses

Heat-inactivated plasma or purified antibodies were prepared in quadruplicate and mixed with 10 TCID_50_ (50 percent tissue culture infective dose) of SIV_mac239_ [22]. In each mixture, 5 µl of diluted sample was incubated with 5 µl of virus. After 45 min incubation at room temperature, each 10 µl mixture was added into 5 x 10^4^ HSC-F cells (macaque T cell line) [23] per well in 96-well plates. Day 10 culture supernatants were harvested and progeny virus production was examined by determining the supernatant reverse transcriptase activity to confirm the absence of neutralizing activity at 1:2.

#### Whole virus ELISA and immunoblotting

SIV virions used for the antigen were prepared by infecting HSC-F cells with SIV_mac239_ at MOI 0.01. Day 7 supernatant was collected and virus particles were purified by centrifugation at 35,000 rpm, 75 min on 20% sucrose in a SW41 rotor (Beckman Coulter), followed by 35,000 rpm, 75 min on 20%-60% sucrose in a SW55 rotor (Beckman Coulter) and 35,000 rpm, 75 min on 20% sucrose in a SW41 rotor. Precipitated SIV virions were diluted in phosphate buffered saline (PBS) and used to coat 96-Well Assay Plates (Becton Dickinson) at a concentration of 100 ng/ml p27 (0.1 ml per well) by overnight incubation at 4 ^°^C. Wells were washed with PBS and blocked with 0.5% bovine serum albumin (BSA)/PBS. Purified anti-SIV immunoglobulin G (IgG) serially diluted in PBS (0.1 ml per well) were incubated for 2 hr at 37 ^°^C. Plates were washed with PBS and virion-bound antibodies were detected with a horseradish peroxidase (HRP)-conjugated goat anti-monkey IgG (H+L) (Bethyl Laboratory) and SureBlue TMB 1-Component Microwell Peroxidase Substrate (KPL). SIV-specific IgG activity in the purified IgG and plasma samples were detected using a Western blotting system for detection of SIV_mac251_ antigens (ZeptoMetrix) according to the manufacturer’s instruction. Samples from animals (R02-006, R04-011, R04-014, and R06-005) with rapid AIDS progression showed lower antibody reactivity.

#### Antibody-dependent cell-mediated virus inhibition (ADCVI) assay

HSC-F cells (1 x 10^5^) serving as MHC-mismatched targets were infected with SIV_mac239_ at MOI 0.001. After adsorption for 6 hr, cells were washed twice with medium and serially-diluted anti-SIV or control antibodies (1.0 or 0.1 mg/ml) were added to the target cells with 4 x 10^5^ effector cells, rhesus peripheral blood mononuclear cells (PBMCs), at an E:T ratio of 4:1 in round-bottomed 96-well plates. Wells of target cells without antibodies or effector cells were set as negative controls. After 7 days of culture, supernatants were collected and measured for their Gag p27 concentrations by ELISA (ABL). The percentage of virus inhibition deriving from ADCVI was calculated as follows: % inhibition = 100x (1 - [p27p/p27c]); where p27p and p27c are the average p27 concentrations in wells with anti-SIV and control antibodies, respectively. Experiments were performed twice in duplicate.

#### Antibody preparation

Ten lots of IgG solutions were prepared from ten chronically SIV_mac239_-infected rhesus macaques without detectable SIV_mac239_-specific NAb responses, respectively. IgG was purified from the plasma after heat-inactivation and filtration by Protein G Sepharose 4 Fast Flow (Amersham) and concentrated by Amicon Ultra 4, MW50000 (Millipore) to 30 mg/ml. Purified IgG solutions were confirmed negative for SIV_mac239_-specific neutralizing activity. Three lots prepared from three macaques (R06-007, R01-009, and R03-005) were mixed to obtain the IgG inoculums for passive non-NAb immunization. Five lots of IgG solutions were also prepared from five chronically SIV_mac239_-infected rhesus macaques with detectable SIV_mac239_-specific NAb responses, respectively. Control IgG (CAb) was prepared from pooled plasma of non-infected rhesus macaques.

#### Animal experiments

Burmese rhesus macaques (*Macaca mulatta*) were challenged intravenously with 1,000 TCID_50_ of SIV_mac239_. For passive immunization, animals were intravenously administered with 300 mg of anti-SIV non-NAb IgG or control IgG at day 7 post-challenge. The determination of major histocompatibility complex class I (MHC-I) haplotypes was based on the family study in combination with the reference strand-mediated conformation analysis (RSCA) of *Mamu-A* and *Mamu-B* genes and detection of major *Mamu-A* and *Mamu-B* alleles by cloning the reverse transcription (RT)-PCR products as described previously [24–27]. Data on control macaques R10-005, R10-008, and R10-001 have previously been reported [28].

#### Measurement of virus-specific T-cell responses

Virus-specific CD8^+^ T-cell responses were measured by flow-cytometric analysis of gamma interferon (IFN-γ) induction as described previously [29]. PBMCs were cocultured with autologous herpesvirus papio-immortalized B-lymphoblastoid cell lines (B-LCLs) pulsed with overlapping peptide pools spanning the SIV_mac239_ Gag, Pol, Vif, Vpx, Vpr, Tat, Rev, Env, and Nef amino acid sequence. Intracellular IFN-γ staining was performed using CytofixCytoperm kit (Becton Dickinson). Fluorescein isothiocianate-conjugated anti-human CD4, Peridinin chlorophyll protein-conjugated anti-human CD8, allophycocyanin-conjugated anti-human CD3 and phycoerythrin-conjugated anti-human IFN-γ antibodies (Becton Dickinson) were used. Specific T-cell levels were calculated by subtracting non-specific IFN-γ^+^ T-cell frequencies from those after SIV-specific stimulation. Specific T-cell levels less than 100 cells per million PBMCs are considered negative.

#### Sequencing

Viral RNAs were extracted using High Pure Viral RNA kit (Roche Diagnostics) from macaque plasma obtained at around 1 year after challenge. Fragments of cDNAs encoding SIV_mac239_ Env were amplified by nested RT-PCR from plasma RNAs and subjected to direct sequencing by using dye terminator chemistry and an automated DNA sequencer (Applied Biosystems). Predominant non-synonymous mutations were determined.

#### Statistical analysis

Statistical analysis was performed by Prism software version 4.03 (GraphPad Software, Inc.). Comparison of viral loads, peripheral blood CD4^+^ T-cell counts, peripheral blood central memory CD4^+^ T-cell frequencies, and the number of non-synonymous mutations in Env-coding regions between non-NAb-infused and control animals was performed by nonparametric Mann–Whitney U test with significance levels set at *p* < 0.05. 

## Results

### *In vitro* virion binding and ADCVI activity of SIV-specific non-NAbs

**Figure 1:**
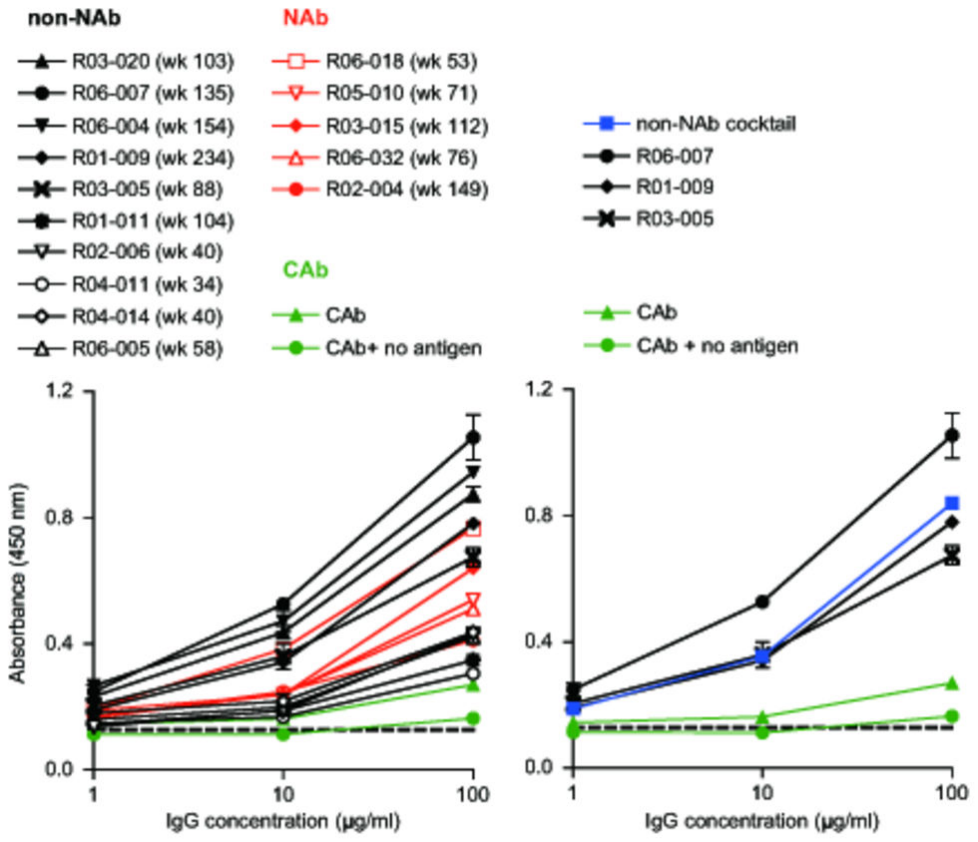
Binding properties of IgGs to SIV virions. Polyclonal IgGs purified from macaque plasma were subjected to whole virus ELISA using purified SIV_mac239_ virions as the antigen. Results on ten IgG lots derived from ten macaques without detectable neutralizing activity (non-NAbs; black lines), five with neutralizing activity (NAbs; red), and a control IgG (CAb; green) are shown in the left panel. Results on the non-NAb cocktail and three non-NAb lots composing the cocktail are in the right. The dotted line represents background absorbance. Time points of plasma sampling are shown in parentheses following the macaque IDs. A representative result, means and SDs of duplicate samples, from two experiments is shown.

**Figure 2:**
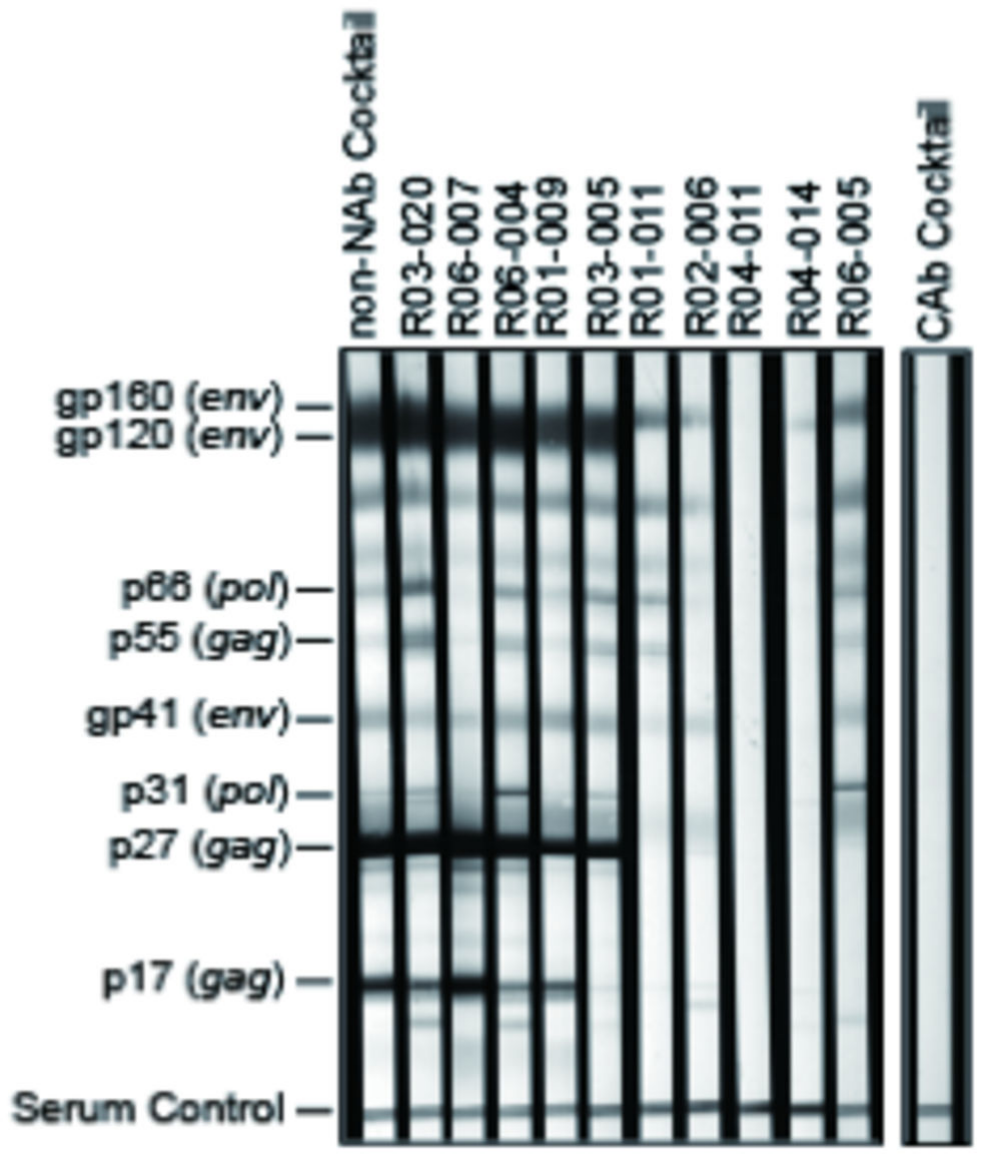
Binding properties of IgGs to SIV antigens. The non-NAb cocktail, ten non-NAb IgG lots derived from ten macaques, and CAb were subjected to immunoblotting (ZeptoMetrix). A representative result from two experiments is shown.

Ten lots of polyclonal IgG were prepared from plasma of ten chronically SIV_mac239_-infected, NAb-negative rhesus macaques, respectively. SIV_mac239_-binding capacity was screened by whole virus ELISA using virions purified from culture supernatants of SIV_mac239_-infected HSC-F cells (a macaque T-cell line) (Figure 1). The measured absorbance was proportionate with Env gp120 and Gag p27 reactivity examined by immunoblotting (Figure 2). Polyclonal IgG lots from three macaques (R06-007, R01-009, and R03-005) with intermediate to high virion-binding capacity, although what percentage of IgGs was SIV-specific are unknown, were pooled and further used as a non-NAb cocktail for passive immunization, whose virion-binding characteristics were also confirmed (Figure 1).

**Figure 3:**
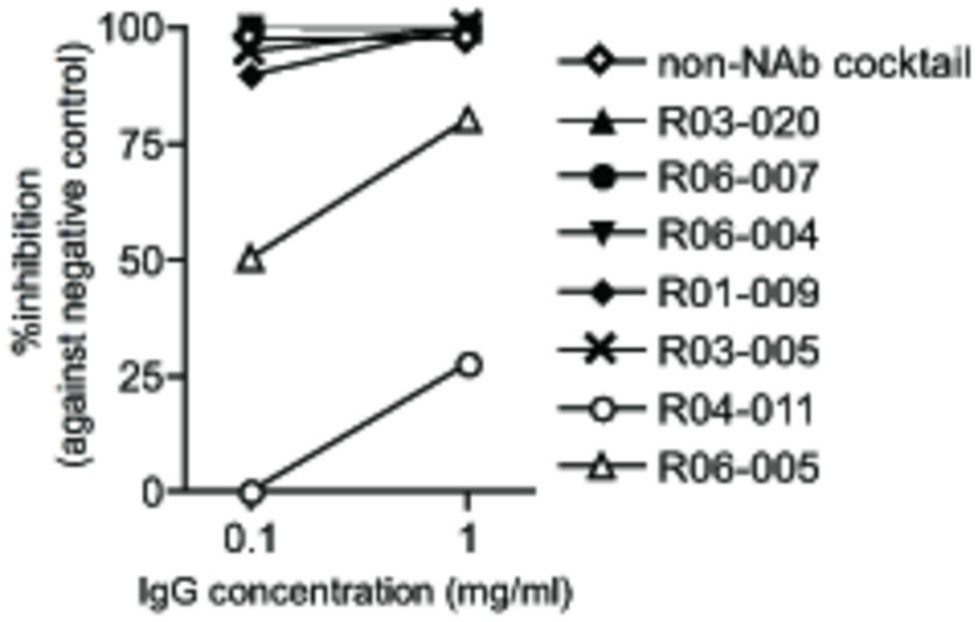
ADCVI activity of the non-NAb cocktail and non-NAb IgG lots. The reduction in SIV p27 concentration in the supernatant from SIV-infected cell culture with non-NAbs compared to that without antibodies is shown. A representative result, means of duplicate samples, from two experiments is shown.

To examine the *in vitro* virus-suppressive activity of the non-NAb cocktail, ADCVI activity was evaluated using PBMCs as effectors and MHC-mismatched macaque HSC-F cells as infected targets (Figure 3). IgG lots with high virion-binding capacity showed high ADCVI activity, whereas those from macaques R04-011 and R06-005 with limited reactivity in ELISA and western blot exhibited low ADCVI activity. These results suggest that ADCVI activity is proportionate with overall virion binding. The non-NAb cocktail exerted more than 97% inhibitory activity even at 0.1 mg/ml IgG concentration. A 1.0 mg/ml IgG concentration approximates an estimated *in vivo* antibody concentration immediately after passive immunization (300 mg IgG in 300 ml body fluid), implying that the observed ADCVI activity is likely to occur *in vivo* after passive immunization.

### *In Vivo* Effect of Non-NAb Passive Immunization in SIV Infection

Having confirmed the *in vitro* anti-viral property of the non-NAb cocktail, we performed the post-infection passive immunization. Five rhesus macaques were challenged intravenously with SIV_mac239_ followed by passive immunization with the non-NAb cocktail (300 mg IgG) at day 7 post-challenge. When we previously passively immunized rhesus macaques with polyclonal antibodies having anti-SIV neutralizing activity by this regimen (300 mg IgG i.v. at day 7), enhanced virus uptake by DCs, subsequent augmentation of SIV-specific CD4^+^ T-cell responses, enhancement of *in vitro* virus-suppressive activity in CD8^+^ cells, and set-point viremia control were observed [20,21]. The current passive immunization experiment contrasts this previous report by infusion of polyclonal antibodies with comparable SIV virion-binding capacity and ADCVI activity without anti-SIV neutralizing activity. The moment of passive immunization (day 7) also recapitulates the first time frame to detect anti-HIV/SIV antibodies after infection [30,31]. Six animals consisting of two without passive immunization and four with control IgG infusion at day 7 after SIV_mac239_ challenge were used as controls.

To examine the abundance of non-NAbs after infusion and *de novo* virus-specific antibody induction, plasma reactivity against SIV antigens was measured by immunoblotting (Table 1). SIV Env-specific antibodies were detected at week 1.5 post-infection exclusively in the non-NAb-infused animals. High reactivity in plasma in these animals resided up to week 3 post-infection. *De novo* induction of SIV-specific antibodies was comparably observed in both the non-NAb-infused and control groups from week 5 to week 12 post-infection. Collectively, the passive non-NAb immunization resulted in systemic distribution of SIV Env-specific antibodies around peak infection.

**Figure 4:**
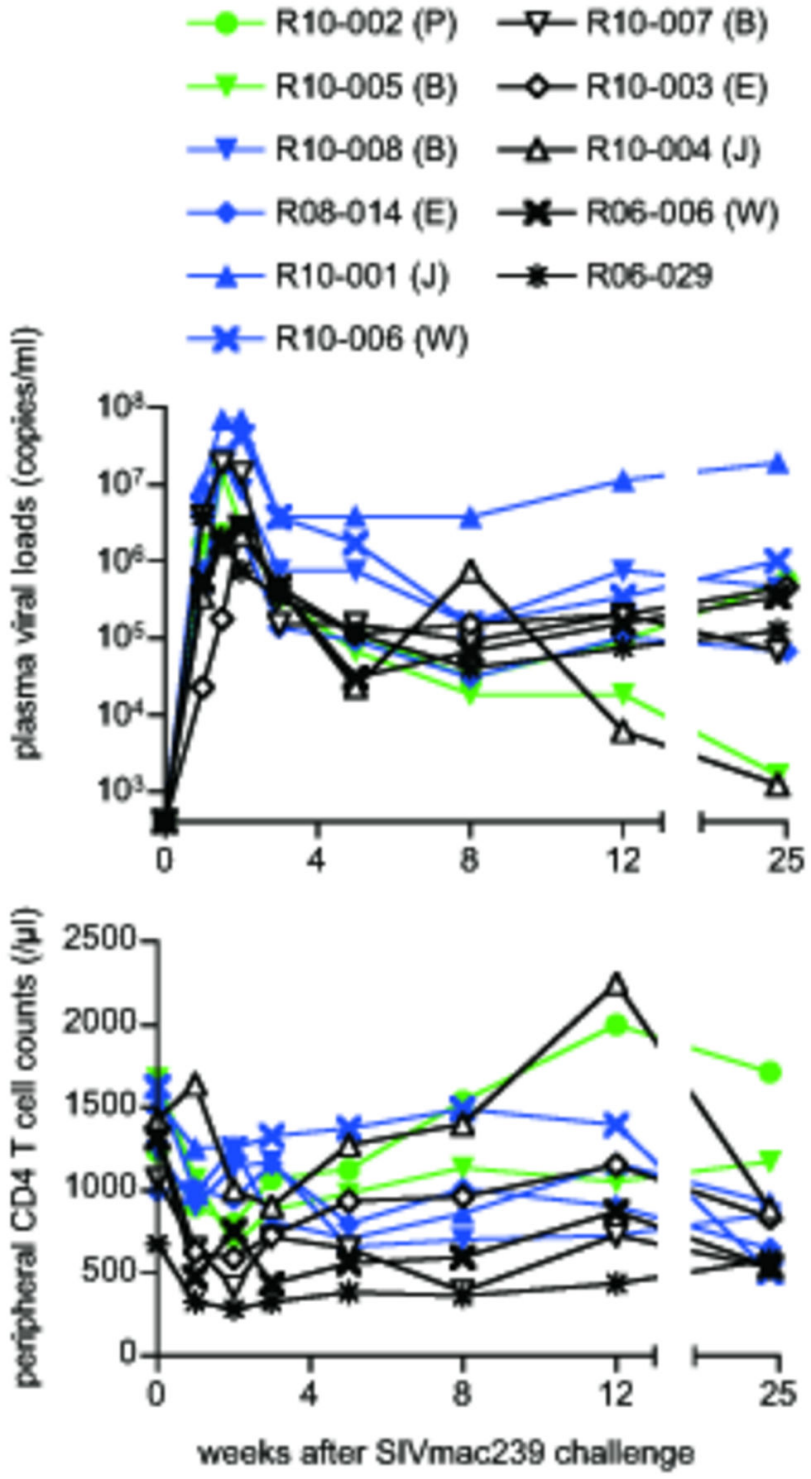
Passive non-NAb immunization in SIV infection. Upper panel: plasma viral loads after SIV_mac239_ challenge (SIV RNA copies/ml in plasma) in two unimmunized (green lines), four control IgG-immunized (blue), and five non-NAb-immunized macaques (black). Viral loads were determined as described previously [25]. The lower limit of detection is approximately 4 x 10^2^ copies/ml. MHC-I haplotypes determined in individual animals are shown in parentheses as follows: B, haplotype *90-120-Ib*; E, *90-010-Ie*; J, *90-088-Ij*; P, *89-002-Ip*; W, *89-075-Iw*. Lower panel: peripheral CD4^+^ T-cell counts after SIV_mac239_ challenge. No significant difference in viral loads or CD4^+^ T-cell counts was observed between non-NAb-immunized and control animals.

**Figure 5:**
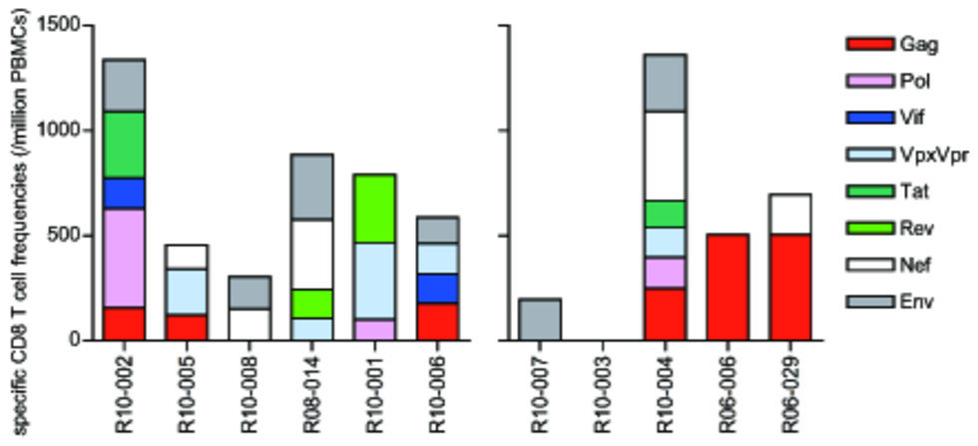
SIV antigen-specific CD8^+^ T-cell responses. SIV Gag-, Pol-, Vif-, Vpx/Vpr-, Tat-, Rev-, Nef-, and Env-specific CD8^+^ T-cell responses were measured by detection of antigen-specific IFN-γ induction using PBMCs at weeks 26-30 post-challenge.

**Table 1:** SIV-specific antibody responses in plasma after SIV infection.

macaques	regimens^a^		plasma antibody responses^b^			
		wk 1	wk 1.5	wk 3	wk 5	wk 12
R10-002	-	-	-	-	+	++++
R10-005	-	-	-	-	+	++++
R10-008	CAb	-	-	-	++	++++
R08-014	CAb	-	-	+	++	++++
R10-001	CAb	-	-	-	+	+
R10-006	CAb	-	-	-	+	++++
R10-007	non-NAb	-	+++	++	++	++++
R10-003	non-NAb	-	++++	++	++	++++
R10-004	non-NAb	-	+++	++	+	++++
R06-006	non-NAb	-	++++	++	+	++++
R06-029	non-NAb	-	+++	++	++	++++

^a^ Animals received no passive immunization (-), passive CAb immunization (CAb), or passive non-NAb immunization (non-NAb) at day 7 after SIV challenge. ^b^ Antibody responses were detected using a commercial Western blotting system (ZeptoMetrix). + Gag p27-positive; ++ Gag p27 and Env gp160-positive; +++ Gag p27, Env gp160, and one other Gag/Pol/Env-derived antigen-positive; ++++ Gag p27, Env gp160, and two or more other Gag/Pol/Env-derived antigen-positive.

All five macaques infused with the non-NAbs failed to contain set-point viremia, similar to the six control animals (Figure 4). The non-NAb-infused and control groups exhibited comparable peak and set-point viral loads without significant difference. No significant difference in total CD4^+^ T-cell counts was found throughout the course between these two groups (Figure 4). Peripheral CD95^+^CD28^+^ central memory CD4^+^ T-cell counts at week 12 were also comparable between these two groups (data not shown).

**Figure 6:**
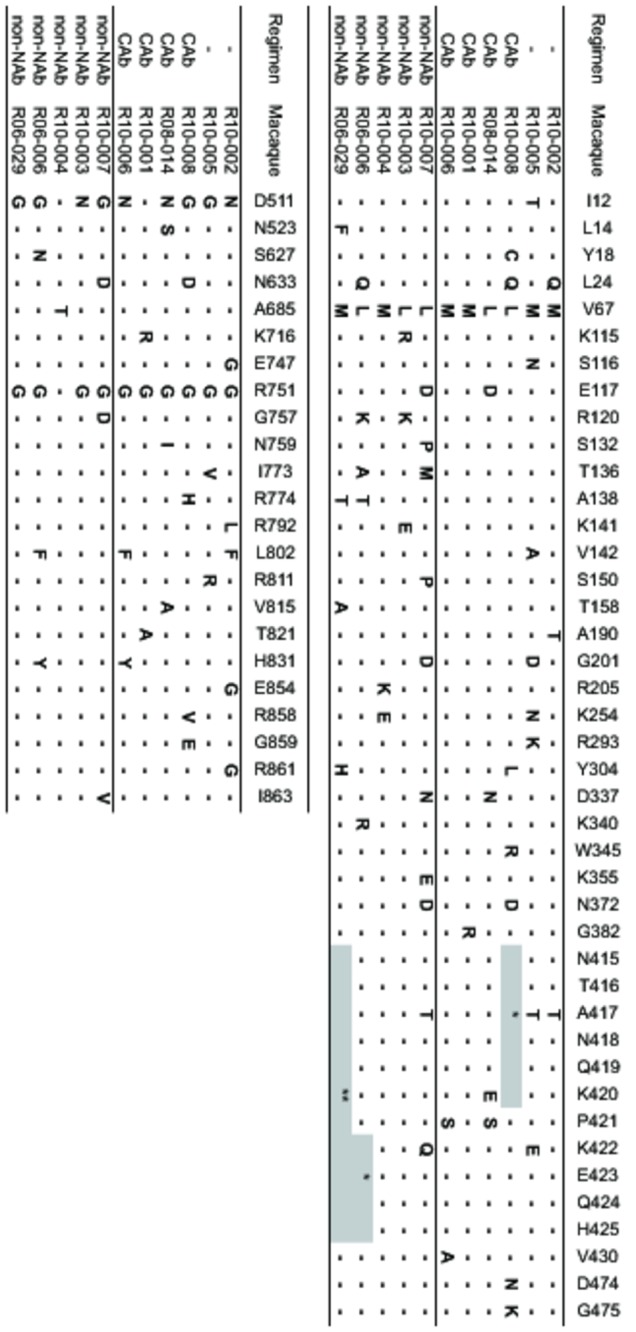
Predominant nonsynonymous env mutations. Viral cDNAs encoding Env were amplified from plasma RNAs obtained at 7-9 months (R10-001, R10-004, and R06-029) or 12 months (other animals) and subjected to sequencing analysis. Amino acid substitutions are shown. The asterisk (*) represents a deletion and the double asterisk (**) represents coexistence of multiple deletion patterns.

Considering our previous study of NAb-triggered SIV control and facilitation of T-cell responses [20,21], we examined SIV antigen-specific CD8^+^ T-cell responses in the chronic phase (Figure 5). Neither the responses to individual antigens nor the summation presented significant difference. Finally, to assess possible selective pressure on SIV by the passive non-NAb immunization, predominant nonsynonymous env mutations in the early phase (at week 12, data not shown) and in the chronic phase were determined (Figure 6). Analysis at week 12 showed only one or two mutations, which were mostly observed also in the chronic phase. Mutations specific for the non-NAb-infused group, such as signs of ADCVI-induced escape [32], were not detected. A slight increase in predominant mutations in the Env V1-coding region was observed in the non-NAb-infused group, although the difference was not statistically significant (*p* = 0.08 by Mann–Whitney U test). Thus, the passive non-NAb immunization at day 7 post-challenge showed no significant impact on SIV replication *in vivo*. 

## Discussion

Whether augmentation of ADCVI without virus neutralizing activity may influence SIV replication control *in vivo* was a major interest in this study. Our results indicate that passive non-NAb immunization does not influence primary SIV replication when administered at early post-infection. In agreement with the limited protective effect observed when non-NAbs were administered locally on mucosa before virus challenge [16], systemic distribution of non-NAbs post-infection did not correlate with suppression of SIV replication, despite antiviral activity of the non-NAbs observed *in vitro*.

For assessment of antibody binding affinity to antigens on virions, we utilized purified SIV virions instead of recombinant Env proteins [33] as the antigen for ELISA under detergent-free conditions. Virion-binding characteristics of antibodies showed a similar trend with ADCVI activity, as seen in other studies [13]. However, the non-NAb infusion did not result in SIV control *in vivo*, which consequently proposes the following notions. 

First, this study indicates that augmentation of non-NAb-derived virus-suppressive activity does not alter SIV control course once infection is achieved. While previous studies on immunized macaques indicated inverse correlation between ADCC or ADCVI activity at virus challenge and acute plasma viremia [11–14], the degree of non-NAb contribution by itself was not clear since vaccination elicited multiple immune responses. In other reports, intracutaneously infused non-NAbs did not exert protection in neonatal macaques [15] and mucosal non-NAbs showed limited protective activity [16]. In coherence, even massive systemic distribution of non-NAbs at peak infection did not impact viral replication in the present study. Thus, antiviral non-NAb responses do not suffice for counteracting establishment of set-point viremia, although these responses may partially influence viral replication in the chronic phase, as indicated by a previous report showing that CD20 depletion in chronic SIV infection can result in accelerated viremia and disease course [34], similar to rapid progressors [35]. Taken together, our results suggest that non-NAbs may withhold a limited role in impeding virus spread *in vivo* in HIV/SIV infections, unlike in other chronic viral infection models [36].

Second, this study indicates the requirement of neutralizing activity of antibody for the suppression of primary SIV replication by passive NAb immunization post-infection, as observed in our previous study [20]. The NAb-triggered SIV control has been suggested to be attributed to antibody-mediated virion uptake by DCs and enhanced T-cell priming [21], which can also occur in the non-NAb-infused animals. Thus, the control failure in non-NAb-infused macaques implies that augmentation of antigen presentation alone may be insufficient for primary SIV control and that reduction of infectious virus burden and CD4^+^ T-cell preservation is important for any immune augmentation [20,37,38].

In conclusion, the post-infection passive non-NAb immunization did not result in primary SIV control in a rhesus macaque AIDS model. Our results suggest that virion binding and ADCVI activity with lack of virus neutralizing activity in the acute phase are insufficient for giving an impact on primary HIV/SIV replication. Further sophistication of local and targeted induction of functional non-NAb responses may be required to impact HIV/SIV replication *in vivo*. 
